# Topological-insulator-based terahertz modulator

**DOI:** 10.1038/s41598-017-13701-9

**Published:** 2017-10-18

**Authors:** X. B. Wang, L. Cheng, Y. Wu, D. P. Zhu, L. Wang, Jian-Xin Zhu, Hyunsoo Yang, Elbert E. M. Chia

**Affiliations:** 1 Division of Physics and Applied Physics, School of Physical and Mathematical Sciences, Nanyang Technological University, Singapore, 637371 Singapore; 2 Department of Electrical and Computer Engineering, National University of Singapore, Singapore, 117576 Singapore; 3 School of Applied Sciences, RMIT University, Melbourne, Victoria, 3001 Australia; 4 Theoretical Division and Center for Integrated Nanotechnologies, Los Alamos National Laboratory, New Mexico, 87545 USA

## Abstract

Three dimensional topological insulators, as a new phase of quantum matters, are characterized by an insulating gap in the bulk and a metallic state on the surface. Particularly, most of the topological insulators have narrow band gaps, and hence have promising applications in the area of terahertz optoelectronics. In this work, we experimentally demonstrate an electronically-tunable terahertz intensity modulator based on Bi_1:5_Sb_0:5_Te_1:8_Se_1:2_ single crystal, one of the most insulating topological insulators. A relative frequency-independent modulation depth of ~62% over a wide frequency range from 0.3 to 1.4 THz has been achieved at room temperature, by applying a bias current of 100 mA. The modulation in the low current regime can be further enhanced at low temperature. We propose that the extraordinarily large modulation is a consequence of thermally-activated carrier absorption in the semiconducting bulk states. Our work provides a new application of topological insulators for terahertz technology.

## Introduction

Terahertz (THz) technology has been well developed in the past several decades with applications spanning from time-domain spectroscopy^1^, to public security^2^, medical imaging^3^, and high speed communications^4^. High performance THz components including sources^5^, detectors^6^ and modulators^7^ are urgently needed to promote further THz technology applications. In an advanced THz system, modulators can be used to actively control the amplitude, phase, and spectrum of the THz wave. THz modulators based on semiconductors and metamaterials have been demonstrated to control the carrier concentration and thus the optical response of semiconductors by electrical or optical doping^8–12^. Moreover, some phase transition materials, such as VO_2_ and superconductors, have been applied and incorporated with metamaterials to thermally modulate the electric conductivity^13–16^. However, conventional thermal-controlled modulators have integration issues with current semiconductor techniques. Recently, it was found that graphene-based modulators have superior performances due to its special band structure with linear dispersion and density of states close to the Fermi energy^17–20^. In particular, a broadband modulation depth of up to 93% based on graphene/ionic-liquid/graphene sandwich structure has been achieved^20^.

Topological insulators (TIs), which are considered as three dimensional analogies of graphene, possess linear Dirac-like states in the insulating bulk gap^21,22^. In contrast to graphene, the strong spin-momentum locking of helical surface states can enable the conversion of charge current into spin current^23^ which offer promising applications in electronic and optoelectronic devices^24–28^. Although the existence of surface states at room temperature has been confirmed by angle-resolved photoemission spectroscopy (ARPES) results^29–31^, the surface states are always contaminated by the residual conductivity in the bulk arising from the presence of intrinsic impurities^32,33^. Alternatively, as narrow bandgap semiconductors, e.g., Bi_2_Se_3_ and Bi_2_Te _3_ with bulk gap of ∼300 and 150 meV, respectively^34,35^, TIs are known to be excellent thermoelectric materials^36,37^ and have potential applications at room temperature^25^. Recently, Bi_1.5_Sb_0.5_Te_1.8_Se_1.2_ (BSTS), one of the most insulating topological insulators has been characterized by Terahertz Time-Domain Spectroscopy (THz-TDS), which indicated the presence of an impurity band about 30 meV below the Fermi level^38^. The pronounced temperature dependence of low energy absorptions may be exploited to construct a THz modulator.

Here, we demonstrate a current-driven THz intensity modulator using BSTS crystal. High modulation depth over a broadband THz region is obtained with applied in-plane current. We also show that the THz modulation could be further enhanced at cryogenic temperatures. Moreover, we confirm that the large modulation arises from the thermal-activated free carriers in the semiconducting bulk state.

## Results

**Figure 1:**
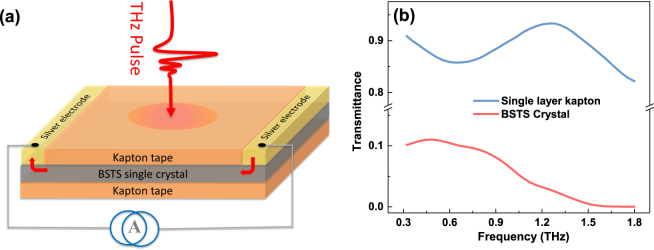
(**a**) Schematic illustration of Kapton/BSTS/Kapton sandwich-structure THz modulator. (**b**) Transmittance of 30-$\mu$m-thick BSTS crystal and single layer Kapton tape at room temperature.

**Figure 2:**
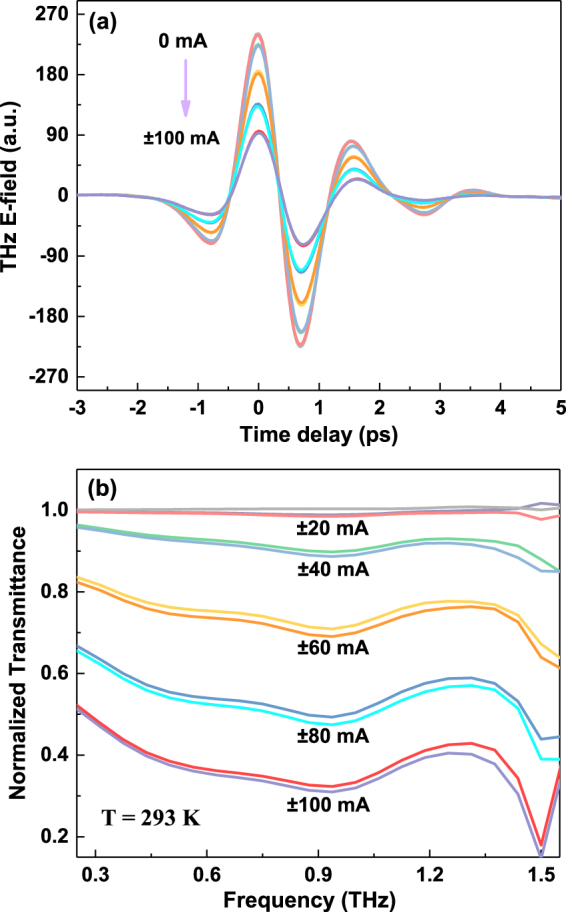
(**a**) Measured THz waveforms transmitted through the device under different bias current from 0 to 100 mA in a step of 20 mA at room temperature. (**b**) The corresponding THz transmittance spectra normalized to the spectrum at zero bias.

The device studied, as shown in Fig. 1(a), is a sandwich structure consisting of a BSTS crystal and two layers of Kapton tapes. Figure 1(b) shows the transmittance of 30-$\mu$m-thick BSTS single crystal and single layer Kapton tape, about 10% and 90%, respectively. The transmittance of BSTS almost drops to zero above 1.5 THz. This could be explained by the existence of an optical phonon mode at 1.9 THz^38,39^, resulting in the strong absorption in the transmittance spectrum above 1.5 THz. Figure 2(a) shows the measured transmitted THz waveform in the time domain as a function of in-plane current at room temperature. The peak amplitude of electric field decreases significantly with the applied DC current tuned from 0 to 100 mA. The attenuation of THz peak are the same at both positive and negative current (negative means reversing the direction of in plane current). Also, no obvious peak shift was observed in the THz pulses.

**Figure 3:**
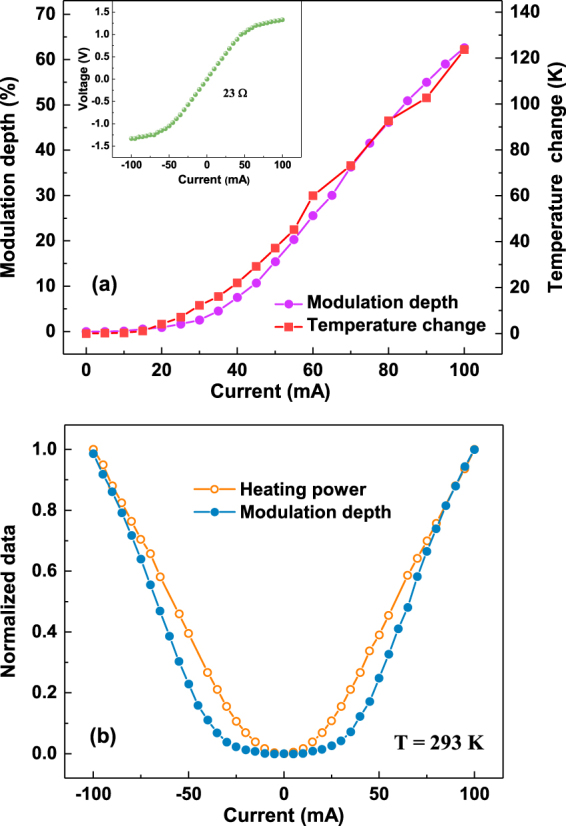
(**a**) Modulation depth at 0.5 THz and temperature change of the device and (**b**) Normalized modulation depth and heating power as a function of applied in plane current at room temperature. Inset: Current–Voltage (I-V) characteristic of the modulator at room temperature.

By Fast Fourier Transformation (FFT) of the time domain pulses, the corresponding THz amplitude spectra are obtained. These spectra are normalized by a reference spectrum obtained from the same device without applying current, as shown in Fig. 2(b). The normalized strength of the THz electric field decreases with increasing bias current at the frequency range from 0.3 to 1.4 THz, above which the signal is unreliable due to the strong absorption. To verify the performance of this THz modulator more clearly, the relative change in the amplitude of transmittance is used to define the modulation depth: $MD=|t(I)-t\mathrm{(0)}|/t\mathrm{(0)}$, where *t(0)* and *t(I)* are the electric field transmittance of the device under zero and non-zero biased current, respectively. A relative flat modulation depth is achieved in the 0.3–1.4 THz frequency range at various bias current, as indicated in Fig. 2(b). Increasing the magnitude of the current from 0 to 100 mA decreases the relative transmittance significantly, achieving a maximum modulation depth of 62% (at 0.5 THz, peak position of spectra) at the highest bias current, as illustrated in Fig. 3(a).

A three-dimensional topological insulator has metallic surface sate in the insulating bulk energy gap. Thus one should expect the surface states to dominate the electric transport. However, as mentioned above, due to the free carriers in the bulk, the contribution of surface states is difficult to detect. In other words, the electric transport at room temperature is dominated by the semiconducting properties of bulk states. For our BSTS sample, an impurity band lies ∼30 meV below the Fermi level with a bulk gap of 0.25 eV^38,40,41^. Current-Voltage (I-V) measurements, as shown in the inset of Fig. 3(a), further confirms the Schottky character of the modulator. The symmetric behavior of the I-V characteristic may be due to the formation of two back-to-back Schottky diodes at the interfaces of BSTS and silver paste. A flow of electric current through the two electrodes will cause a Joule heating effect. As the current increases, the device is heated, and the corresponding rise in temperature of the device is ∼124 K for the maximum current amplitude of 100 mA. A larger thermal energy causes more electrons to be excited from the impurity band to the Fermi level in BSTS, which then results in larger absorption of THz radiation by these electrons via intraband transition. The temperature change of the device surface is also plotted in Fig. 3(a), showing excellent agreement with THz modulation depth at 0.5 THz. This agreement is a consequence of the fact that, in a dielectric slab, the change in real part of the optical conductivity relative to the zero-current conductivity, ${\rm{\Delta }}{\sigma }_{1}\equiv {\sigma }_{1}(I)-{\sigma }_{1}(I\,=\,\mathrm{0\ }mA)$, is proportional to both (1) ${\rm{\Delta }}E/{E}_{0}$ ^42^, which is the modulation depth, as well as (2) temperature change of the device to a first approximation according to ${\rm{\Delta }}{\sigma }_{1}\approx (d{\sigma }_{1}/dT){\rm{\Delta }}T$. Moreover, the temperature change of the device should be proportional to the heating power (voltage multiplied by current). Therefore the similar current dependence between the normalized modulation depth and heating power, as shown in Fig. 3(b), provide additional evidence that the large modulation depth obtained here is related to thermal heating effect.

The THz conductivity of BSTS was studied by Tang *et al*., from which, both the low-frequency ${\sigma }_{1}(\omega$ = 0.4 THz) and the square of plasma frequency ${\omega }_{p}$ could be well described by a thermally-activated hopping model^38^. The plasma frequency is related to the carrier density $n$ via ${\omega }_{p}^{2}=n{e}^{2}/{\varepsilon }_{0}{m}^{\ast }$, where the *e* is elementary charge, ${\varepsilon }_{0}$ is the permittivity of free space, and the ${m}^{\ast }\,=\,0.32{m}_{e}$ is the effective mass of the conduction carrier^41^. Based on the fitting parameters from Tang *et al*., the carrier concentration at 293 K and 417 K are estimated to be $8.5\times {10}^{17}$ and $1.44\times {10}^{18}$ cm^−3^, respectively^38^, which is consistent with the Fourier transform infrared spectroscopy results^43^. Therefore the thermally-induced carrier density is about $6\times {10}^{17}$ cm^−3^ at room temperature under 100 mA bias current. Thus the relative change of the carrier density under 100 mA bias current is roughly 70%, comparable to the modulation depth at room temperature. These thermally-induced carriers absorb the THz wave, leading to the significant decrease of transmission of THz wave.

**Figure 4:**
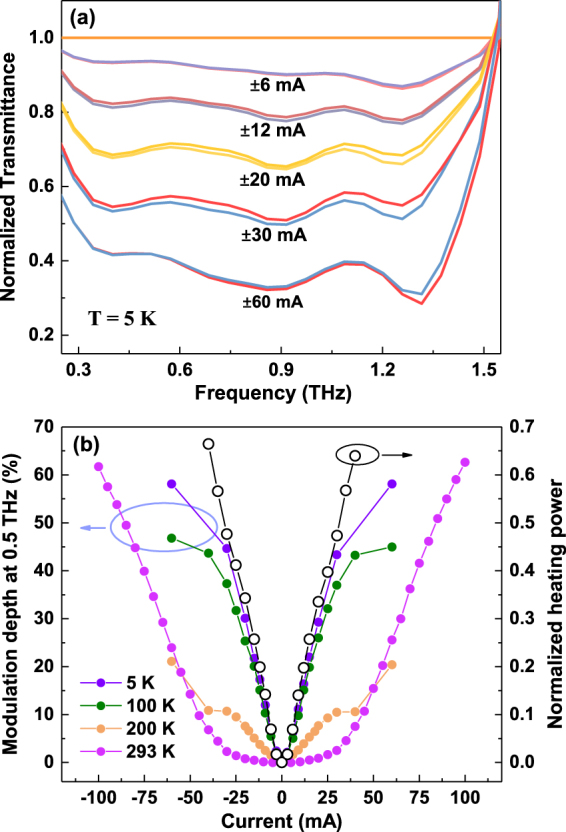
(**a**) Normalized THz transmittance spectra under various bias current at 5 K. (**b**) Modulation depth at 0.5 THz for various temperatures (filled circle) and normalized heating power at 5 K (open circle) versus the applied in-plane current.

After identifying the thermal origin of the large modulation effect, we measure the THz response of the device under various bias current at temperatures down to 5 K. The normalized transmittance spectra under different bias current at 5 K is shown in Fig. 4(a). The modulation is significantly enhanced than that at room temperature, e.g., 6 mA bias current could lead to a modulation depth of 10%. Figure 4(b) shows the modulation depth at 0.5 THz under various temperatures. We see that a higher bias current is needed to achieve the same modulation depth at 5 K with increasing temperature. In the low-bias current range, the modulation depth is much higher at lower temperature under the same current, especially below 100 K, increasing linearly with applied current. As mentioned above, the BSTS bulk sample shows typical semiconductor behavior. The resistivity of the device, derived from the I-V curve at low biased current, increases from 23 ${\rm{\Omega }}$ at room temperature to 453 ${\rm{\Omega }}$ at 10 K. Higher resistivity means stronger heating effect. Moreover, the thermal conductivity of Kapton tape below 100 K is about two orders of magnitude smaller than that at room temperature^44^, which means the heat generated by Joule heating cannot be removed fast enough by thermal conduction through the Kapton tape. Therefore the higher temperature change induces more thermally-activated carriers, and leads to a larger modulation depth. The consistency between the modulation depth and normalized heating power in this regime at 5 K, as illustrated in Fig. 4(b), again supports the thermal origin of THz modulation. For high bias current range (above 30 mA), the modulation depth at low temperature deviates from the linear behavior, which can be explained by the extremely large heating effect. The BSTS crystal could be heated up to a very high temperature even though the sample holder is still kept at the fixed experimental temperature. At the same time, thermal conductivity of Kapton increases slightly with increasing temperature, resulting in higher equilibrium temperature of the whole device. Consequently, the temperature change tends to saturate, leading to the saturation of modulation depth at higher bias current regime. Note that the maximal bias current at low temperatures is 60 mA, above which the device would be damaged.

One may argue that the large modulation depth at low temperature should be related to the surface states. The transmittance of one surface layer of BSTS is estimated about 98.3%^38^, which is much larger than the transmittance we observed in our data. This suggests that THz absorption even at low temperature is still dominated by the bulk. On the other hand, for a fixed experimental temperature, the transmittance under different current is referenced to that without applying current, according to which the contribution from surface states could be eliminated. Therefore we can conclude that the dramatic increase of modulation depth with increasing in-plane current at low temperature is also related to the semiconducting bulk states.

## Discussion

The carrier concentration in BSTS can be electrically controlled by tuning the temperature of the crystal, making it possible to modulate the terahertz wave through the device. On the basis of this principle, a highly tunable broadband THz intensity modulator based on topological insulator is proposed and experimentally demonstrated. The electric-field modulation depth is about 62% in 0.3–1.4 THz range corresponding to a power modulation depth of ∼85% which is significantly higher than that of most of the previously developed semiconductors-based modulators^8–12^. Although the insulator-to-metal phase transition of VO_2_ can offer a higher modulation depth, the electrical controllability of the device requires a very high voltage^45^. On the other hand, the high modulation depth of our device could be obtained with a bias current of 100 mA or an equivalent bias voltage less than 1.5 V, which is comparable to that of the single-layer graphene-based modulator by ionic liquid gating^20^. Therefore, we can confidently say that the TI-based device could be utilized as a high-efficiency THz intensity modulator.

Besides the modulation depth, the insertion loss is also important for evaluating a modulator. For our sandwich-structure device, the electric field peak attenuation is about 92% at room temperature, which can be diminished by optimizing the sandwich structure. On the one hand, decreasing the thickness of the TI crystal could decrease the free carriers absorption in the bulk. The transmittance of BSTS flake which is mechanically exfoliated from the single crystal pallet increases from 10% to 64% when the thickness decreases from 30 $\mu$m to 1.3 $\mu$m at room temperature, as shown in the Fig. 1(b). On the other hand, using the more THz-transparent thin film capping layers, for example Al_2_O_3_, to instead of the Kapton tape can further minimize the insertion loss.

In conclusion, we demonstrated a proof-of-principle topological-insulator-based THz intensity modulator fabricated by BSTS single crystal, which can be efficiently controlled by a DC current. We observed a maximal modulation depth about 62% for our sandwich-structured device in a wide frequency range from 0.3 to 1.4 THz at room temperature, with current modulation further enhanced at low temperature. We also confirmed the observed large THz modulation to be mainly due to the temperature-tunable carrier population of bulk states. Our results suggest a new application of topological insulators for terahertz technology.

## Methods

### Device fabrication

High-quality BSTS single crystals are synthesized using the modified Bridgeman method, and can be easily cleaved using Kapton tape. Their structure and transport properties have been reported in an early study^40^. The devices are fabricated using a two-step tape method. First, a BSTS flake (~30 *μ*m) is mechanically exfoliated from single crystal pallet using Kapton tape. Second, most of top surface of the BSTS crystal is covered by another Kapton tape, which prevents the decay of the sample in the ambient atmosphere. Kapton tape (typical thickness of ∼70 *μ*m) can remain stable over a broad range of temperatures. Silver paste is used to form two electrodes on the exposed BSTS crystal. Thus a device based on Kapton/BSTS/Kapton sandwich structure is prepared for THz modulation with a clear aperture of ∼6 $\times$ 3 mm^2^ for testing.

### THz-TDS measurements

THz time-domain spectra of the sandwich-structure device were measured by TPS–3000 spectrometer with a frequency range of 0.3–2.7 THz which is incorporated with a Janis ST-100-FTIR continuous flow cryostat in the temperature range from 5 to 400 K. The in-plane current was applied between the two electrodes using a Keithley 2400 sourcemeter operating in direct current mode with the voltage being measured simultaneously. Data collection under different bias currents was initiated after stabilization of the source-drain voltage which typically took about 1–5 seconds. Each trace was averaged from 900 spectra with a scanning frequency of 30 Hz. The temperature of the device under various bias currents at room temperature was measured separately using a FLIR T620 thermal imaging camera at ambient condition.

### Data Availability

All data generated or analysed during this study are included in this published article.
